# Complete Genome Sequence of an African Swine Fever Virus Isolate from Sardinia, Italy

**DOI:** 10.1128/genomeA.01220-16

**Published:** 2016-11-17

**Authors:** Fredrik Granberg, Claudia Torresi, Annalisa Oggiano, Maja Malmberg, Carmen Iscaro, Gian Mario De Mia, Sándor Belák

**Affiliations:** a Department of Biomedical Sciences and Veterinary Public Health, Swedish University of Agricultural Sciences (SLU), Uppsala, Sweden; b Istituto Zooprofilattico Sperimentale dell’Umbria e delle Marche (IZS-UM), Perugia, Italy; c Istituto Zooprofilattico Sperimentale della Sardegna (IZS-SA), Sassari, Italy; d SLU Global Bioinformatics Centre, Department of Animal Breeding and Genetics, Swedish University of Agricultural Sciences (SLU), Uppsala, Sweden

## Abstract

Previous genetic characterization of African swine fever virus isolates from the Italian island of Sardinia, where the virus has been present since 1978, has largely been limited to a few selected genomic regions. Here, we report the complete genome sequence of the isolate 47/Ss/08 collected during an outbreak in 2008.

## GENOME ANNOUNCEMENT

African swine fever (ASF) is a highly contagious and devastating disease of pigs, which is enzootic in many African countries and on the Italian island of Sardinia (1). The etiological agent, ASF virus (ASFV) (Asfarviridae*, Asfivirus*), is an enveloped virus with a linear double-stranded DNA (dsDNA) genome of 170 to 190 kbp, flanked by inverted terminal repeats (ITRs). Based on nucleotide sequencing of the p72 capsid protein gene, 22 genotypes (I to XXII) have been distinguished, but additional genomic regions have been suggested for increased resolution (2, 3). Genetic analysis of ASFV isolates from Sardinia revealed that they all belong to the p72 genotype I, with only minor variations within the B602L and the EP402R genes (4, 5). To better distinguish between these closely related isolates, comparative analysis of near-full-length or complete viral genome sequences is required.

The 47/Ss/08 isolate was collected in 2008 during an outbreak in the Sardinian province of Sassari. Viral DNA was purified from a blood sample of a viremic pig (6). Initial sequencing was performed on an Illumina MiSeq using the Nextera XT kit for library construction and the V3 reagent kit for 300-bp paired-end reads. The reads were *de novo* assembled using MIRA version 4.0.2, with default settings. BLASTn comparison of contigs with the NCBI nt database identified Benin 97/1 (accession no. AM712239) as the most similar reference genome. The abundance of ASFV in the DNA sample was estimated by mapping quality-filtered and trimmed reads to the reference genome using the FASTX-Toolkit and Bowtie 2 (7). This revealed that 67.80% of the reads belonged to ASFV and also identified regions with low mapping quality or coverage. These regions either sufficiently diverged from the reference to prevent mapping or contained repetitive elements, such as tandem repeats. The Pacific Biosciences (PacBio) RSII platform was used to generate long-read sequence data that potentially could reduce the assembly complexity (8). A total of 56,320 PacBio subreads, with a mean mapped read length of 2.44 kb, were generated from a 2.5-kb library on a single-molecule real-time (SMRT) cell. The SMRT Analysis system version 2.3.0 was used for *de novo* assembly, resulting in three major contigs. These were combined with the MiSeq contigs into a single consensus sequence using CodonCode Aligner version 6.0.2, and the ITRs were manually corrected. Regions of low quality were verified by PCR and Sanger sequencing.

The complete genome consists of 184,638 nucleotides and has a mean GC content of 38.5%. Annotation was performed by using the GATU software (9), with selected ASFV annotated genome references from GenBank, and Prokka version 1.11 (10). All annotations were combined and manually curated using the Ugene software package (11). The most similar ASFV sequences at the time of analysis were Benin 97/1 (accession no. AM712239) and E75 (accession no. FN557520), both with 99% identity and between 98 and 99% query coverage, as revealed by BLASTn search against the NCBI nt database. Considering the high genetic similarity, it is reasonable to conclude that 47/Ss/08 belongs to the same virulent subgroup as Benin 97/1 and E75 (12).

### Accession number(s).

The genome sequence of the ASFV isolate 47/Ss/08 has been deposited in GenBank under the accession number KX354450.
